# The Serotonin Transporter Gene Alters Sensitivity to Attention Bias Modification: Evidence for a Plasticity Gene

**DOI:** 10.1016/j.biopsych.2011.07.004

**Published:** 2011-12-01

**Authors:** Elaine Fox, Konstantina Zougkou, Anna Ridgewell, Kelly Garner

**Affiliations:** a Department of Psychology, University of Essex, Colchester, United Kingdom; b Department of Mental Health Sciences, University College London, London, United Kingdom; c Queensland Attention and Control Laboratory, University of Queensland, Queensland, Australia

**Keywords:** Anxiety, attention bias modification, cognitive bias, genetics, GxE interaction, serotonin transporter gene

## Abstract

**Background:**

Attention bias modification (ABM) procedures have been shown to modify biased attention with important implications for emotional vulnerability and resilience. The use of ABM to reduce potentially toxic biases, for instance, is a newly emerging therapy for anxiety disorders. A separate line of gene-by-environment interaction research proposes that many so-called vulnerability genes or risk alleles are better seen as plasticity genes, as they seem to make individuals more susceptible to environmental influences for better and for worse.

**Methods:**

A standard ABM procedure was used with a sample of 116 healthy adults. Participants were randomly assigned to one of two training groups. One received an ABM procedure designed to induce a bias in attention toward negative material, while the other was trained toward positive pictures. Individuals with low- and high-expressing forms of the serotonin transporter gene (5-HTTLPR) were compared.

**Results:**

Those with a low-expression form (S/S, S/Lg, or Lg/Lg) of the 5-HTTLPR gene developed stronger biases for both negative and positive affective pictures relative to those with the high-expression (La/La) form of the gene.

**Conclusions:**

Here, we report the first evidence that allelic variation in the promotor region of the 5-HTTLPR gene predicts different degrees of sensitivity to ABM. These results suggest a potential cognitive mechanism for the gene-by-environment interactions that have been found in relation to the serotonin transporter gene. Variation on this genotype may therefore determine who will benefit most (and least) from therapeutic interventions, adversity, and supportive environments.

The search for vulnerability genes or risk alleles has been central to the field of psychiatric genetics. It now seems that while specific genes are unlikely to be linked in a direct way to psychopathology, they do moderate the impact that the environment has on stress sensitivity (1–3). Evidence for such gene-by-environment (GxE) interactions, in spite of ongoing controversy, has been gaining momentum. Central to this debate is the burgeoning number of studies examining a repeat length polymorphism in the promotor region of the human serotonin transporter gene (*5-HTT*, *SLC6A4*), which has become the most widely studied genetic variant in psychiatry, psychology, and neuroscience (4–9). The short (s) allelic form of the serotonin transporter-linked polymorphic region (5-HTTLPR) is associated with reduced activity of the serotonin transporter, resulting in higher levels of intrasynaptic serotonin (low expression) compared with the long (l) form, which leads to reduced levels of intrasynaptic serotonin (high expression) (3,8).

In 1996, it was reported that the s allele was associated with increased self-reports of trait-anxiety or neuroticism, a personality construct known to be linked with increased risk of depression (10). Then, in 2003, an influential longitudinal study found that carriers of the s allele were indeed at increased risk of depression and suicidality but only if they had experienced serious stressful life events or childhood abuse (11). This classic GxE interaction led to a burgeoning of research that remains controversial (4–6,12). While some meta-analyses find that the GxE effects do not hold up across studies (12), others find that as long as a detailed and comprehensive analysis of stressful life events is documented, the 5-HTTLPR short variant does moderate the impact of life stress on psychopathology (6,7). Thus, when extensive details are taken with regard to life events, such as relationship breakups, etc. in one-to-one interviews, GxE effects are strong, while they are often not detected when such specifics are not obtained (4).

Another factor that may contribute to the difficulty of replication in this field is the possibility that the s allele actually increases sensitivity to the environment in a more general way so that adverse environments will lead to bad outcomes, while positive and supportive environments will lead to benefits. In other words, the s allele may not be a vulnerability genotype so much as a plasticity genotype (13–15). Uher (2) has argued that one explanation as to why so-called risk alleles have been conserved throughout evolution might be because the social context shapes the outcome of these essentially neutral genetic factors. In other words, more malleable neural circuits can lead to negative outcomes under adversity but also hold the potential for positive gains when the environment is supportive. This means that the neural circuits relating to the processing of affective significance, which are controlled to some extent by the serotonergic system, may be sensitized in s-allele carriers (16). The 5-HTTLPR short variant may, therefore, act as a plasticity gene that renders individuals more susceptible to environmental influences for better and for worse (13–15). It is worth noting, however, that negative material has a stronger draw on attention than does positive material (17). This means that attentional biases to negative, especially threat-related, material is generally stronger than biases toward positive information when compared with a neutral baseline. Thus, while plasticity may operate to both negative and positive information, attention will generally be more responsive to the negative.

A separate line of research shows that biases to selectively process threat-related, relative to positive or benign, information is a risk factor for psychopathology. For example, automatic selective biases to direct attention toward negative material better predicts stress reactivity 4 months later, as measured by cortisol response, relative to standardized measures of neuroticism and trait-anxiety (18). It is, therefore, unsurprising that s-allele carriers usually demonstrate increased attentional bias for threat (19–26), which has been confirmed in a recent meta-analysis, and increased amygdala reactivity to threat-related images (27,28). Of particular interest, a recent study shows that s-allele carriers are faster than l homozygotes to pick up fear responses in a fear-conditioning paradigm (29), supporting the notion that people with this genotype are more sensitive to fear-related cues in the environment. Because fear learning is a primary mechanism through which attentional biases for threat develop (30,31), we can speculate that this may be one mechanism through which s-allele carriers acquire a bias toward the more negative aspects of the environment.

New techniques to actively induce or modify attentional biases provide a unique methodology to test the hypothesis that s-allele carriers' heightened sensitivity to threat results in the development of potentially toxic biases that leave them more susceptible to psychopathology. MacLeod *et al.* (32) first demonstrated that selective biases in attention could be modified by a simple computerized technique and that induction of a threat bias leads to increased stress reactivity, whereas the induction of a benign bias leads to a reduction in emotional vulnerability. These findings are important, as they provide evidence for the causal nature of biased attention in stress vulnerability; an experimentally induced bias changes stress reactivity. Their attention bias modification (ABM) technique involved participants being required to identify a nonemotional probe, such as a letter or a symbol, that could appear in one of two locations on the computer screen immediately following the presentation of two words, one of which was negative (e.g., failure, humiliation) and one of which was neutral (e.g., carpet). To train attention toward negative words, the critical probe always appeared in the location previously occupied by a negative word, whereas to induce a benign bias, the probe always appeared in the location previously occupied by a neutral word. Variants of this ABM task have been tested in a range of anxiety disorders and have been shown to reduce threat-related biases and produce marked improvements in clinical symptoms (33–35). Attention bias modification techniques demonstrate that attentional biases are highly plastic and might provide novel treatment strategies for anxiety disorders (35).

The present study presents the first investigation of the hypothesis that carriers of the short variant of the 5-HTTLPR will be more responsive to ABM interventions. We used a novel form of the ABM task that presented only positive and negative pictures, rather than comparing each with a neutral item. The main reason for this was because the wider literature on ABM conflates valence and arousal. Because we wanted to isolate the effects of valence (negative and positive material), we used well-validated pictorial stimuli that were matched for arousal level. This would not have been possible if a neutral control had been included on each trial. Based on previous findings with fear conditioning (29), we expected s-allele carriers to develop stronger biases for threat in an ABM task when compared with those homozygous for the l allele. Moreover, if the s allele really does confer greater sensitivity to the environment for better and for worse, then we would also expect stronger development of a positive bias for pleasant images in people with this genotype. In contrast, if the s allele is better characterized as a vulnerability gene primarily responsive to fear-relevant information, then sensitivity to environmental contingencies should occur only with threat-related stimuli.

## Methods and Materials

### Participants

Participants were recruited from a pre-existing database at the University of Essex if they carried either the low-expression (i.e., S/S, S/Lg, or Lg/Lg) or the high-expression (La/La) variant of the 5-HTTLPR gene. Sixty-two participants with the low-expression and 54 with the high-expression genotype were recruited. None had a prior or current psychiatric diagnosis and all reported taking no medication that might affect their mental ability. All had normal or corrected-to-normal vision and gave written informed consent to participate in the study. For the low-expression group, 31 participants were randomly assigned to an attention training procedure to induce attentional bias toward negative images (negative ABM), while 31 were assigned to a training condition designed to induce bias toward positive images (positive ABM). For the high-expression genotype group, 26 participants were assigned to the negative ABM, while 26 were assigned to positive ABM. Participants were either paid £6 or awarded course credit for taking part in the experiment.

### Genotyping of Serotonin Transporter Polymorphism

For DNA collection, participants provided three to four eyebrow hairs with their root ball intact, which were placed into a labeled 1.5 mL tube and centrifuged. DNA was extracted using the Qiagen (Qiagen GmbH, Hilden, Germany) DNeasy blood and tissue kit according to the manufacturer's instructions, using 180 uL of ATL buffer plus 20 uL of proteinase K for the extraction (both, Qiagen). DNA was eluted in 200 uL AE buffer from the Qiagen columns and stored at −20°C until analyzed. The samples were assayed with a combined polymerase chain reaction (PCR)/restriction digest procedure that enabled the distinguishing of three alleles of the serotonin transporter, a length polymorphism (long and short alleles), and a single nucleotide polymorphism (SNP) within the long allele of the locus. The following two primers were used for the PCR (the forward primer carries a 6-FAM label at the 5′ end):

IDna5HTTP1FF  Fam-CCCAGCAACTCCCTGTACCCCTCCTA
IDna5HTTPA4R  CGCAAGGTGGGCGGGAGGCT

Qiagen Type-It microsatellite PCR mix was used for the PCR amplification, using a final volume of 10 uL. Each PCR contained 2.5 uL DNA (or water for control subjects), 5 uL of 2 × PCR mix, 1 uL Q reagent, 1 uL of primers, and .5 uL of water. The final primer concentration was 200 nmol/L each primer. The PCR mixes were cycled using the following scheme: hot start at 95°C for 5 minutes; 40 cycles of 94°C for 30 seconds, 68°C for 90 seconds, and 72°C for 90 seconds; then a final extension at 60°C for 30 minutes. An aliquot of the PCR products was diluted 1:40 with water, then 1 uL mixed with 9 uL of formamide containing Rox500 GeneScan molecular weight markers (Applied Biosystems, Foster City, California). Samples were analyzed by capillary electrophoresis in an Applied Biosystems 3730 instrument, enabling the distinguishing of the long allele (351 bases) from the short allele (307 bases).

A second aliquot of 2 uL of the PCR products was digested with the HpaII restriction enzyme in a reaction volume of 20 uL, with 1 unit of enzyme, at 37°C for >90 minutes (Invitrogen, Carlsbad, California). Digest products were diluted 1:40 as before, mixed with formamide plus markers, and separated as above. The sizes of bands generated were 259 bases (long allele plus A SNP base), 217 (short allele), and 86 bases (long allele plus G SNP base).

### Materials

Trait anxiety and depression were measured with the Spielberger Trait-State Anxiety Inventory (STAI) (36) and the Beck Depression Inventory-II (37), in addition to a standardized Attentional Control Scale (38).The State-Anxiety scale of the STAI and two 100-mm visual analogue scales (VAS) were also used to assess anxiety and depression both before and after the ABM procedure. Participants indicated their feelings on the dimensions of anxiety and depression by simply placing an X on a 100 mm VAS ranging from 0 (not at all) to 100 (extremely).

### Attention Bias Modification

Stimuli for ABM were presented on a 24-inch Apple Mac OS X Leopard computer (Apple Inc., Cupertino, California) using Superlab software version 4 (Cedrus Corporation, San Pedro, California) and reaction time and error rate responses were recorded from a standard keyboard. Thirty pictures were selected from the International Affective Picture System (IAPS) (39) and gray-scaled using Adobe Photoshop (Adobe, San Jose, California). Fifteen pictures were negatively valenced (picture numbers: 1111, 1112, 1270, 1275, 1280, 1300, 2205, 2490, 2590, 2691, 2692, 2750, 2800, 9180, and 9253) and 15 pictures were positive images (picture numbers: 1440, 1460, 1463, 1500, 1510, 1540, 1590, 1604, 1610, 1710, 2040, 2660, 4641, 4643, and 4660). The two sets differed on the valence of pictures but were matched on arousal. From this set, four positive and four negative pictures were used in phase 1—pretraining—consisting of 128 trials. Each trial started with a fixation cross at center of the screen for 500 milliseconds. Then, a pair of positive and negative pictures appeared one on either side of the fixation point for 500 milliseconds. Immediately after this display, a target—either horizontal (..) or vertical (:) dots—replaced one of the pictures and remained on the screen until response. Participants had to press the “A” button if the dots were vertical and the “L” button if the dots were horizontal. Errors were indicated by a 50-MHz tone. Following the participant's response, a blank screen was presented for 750 milliseconds before a new trial began. There was a 50:50 chance for the target to replace the negative or the positive picture so that any underlying bias toward either type of image could be assessed.

Phase 2 consisted of 648 training trials with a different set of nine positive and nine negative pictures. Everything was identical to phase 1 except that the stimulus valence to target location contingency was 100% rather than 50%. In the negative ABM, the target always replaced the negative picture (100% contingency), while in the positive ABM, the target always replaced the positive picture (100% contingency). Finally, in phase 3 (posttraining), the contingency reverted to 50:50, and four images (two positive and two negative) from phase 1, along with four new images (two positive and two negative), were presented. Otherwise, everything was identical to phase 1 to assess whether there was any change in attentional bias from before to after ABM training.

### Test Procedure

Following written informed consent, each participant filled out the trait questionnaires followed by the pretraining state-anxiety questionnaire and the two VAS ratings. The nature of the computer task was explained. Following 20 practice trials, participants were then left to complete all three phases of the computerized ABM procedure. When finished, they again completed the state-anxiety questionnaire and the two VAS scales.

### Data Analysis

For each participant, the mean correct reaction time (RT) was computed for each cell of the design by first removing any trials with errors and then removing RTs that were less than 200 milliseconds or greater than 2000 milliseconds (3.1% of data). Preliminary analysis revealed that overall RTs did not differ between the low- and high-expression genotype groups (mean = 750 milliseconds for both). To simply the analysis and presentation of results, an attentional bias index (AB index) was computed by subtracting the mean correct RT on trials where the target appeared in the location of a positive image from the mean correct RT on trials where the target appeared in the location of a negative image. Thus, a numerically positive AB index (e.g., +40 milliseconds) indicates vigilance for affectively positive images (or avoidance of negative images), whereas a numerically negative score (e.g., −52 milliseconds) indicates vigilance for negatively valenced IAPS pictures (or avoidance of positive images). Three separate index scores were computed: AB-pre was attentional bias before training, while AB-old and AB-new were the attentional bias indices found after training for old IAPS images (i.e., those that had been presented in phase 1) and for new IAPS images (i.e., those that had not been presented earlier in the study). Data were analyzed by means of a 2 (genotype group: low expression, high expression) × 2 (ABM group: negative ABM, positive ABM) × 3 (bias index: AB-pre, AB-old, AB-new) analysis of variance with the AB index as the dependent variable. The impact of ABM on mood was assessed by a series of 2 (genotype group: low expression, high expression) × 2 (ABM group: negative ABM, positive ABM) × 2 (session: before training, after training) analyses of variance with state-anxiety and VAS ratings of anxiety and depression as dependent variables.

## Results

Male and female subjects were distributed equally between the low-expression (male subjects = 55%, *n* = 34) and the high-expression (male subjects = 56%, *n* = 30) genotype groups, which were comparable on the STAI trait-anxiety [mean for low expression = 40.08, SD = 10.6; mean for high expression = 39.3, SD = 8.6, *t*(114) < 1], Beck Depression Inventory-II [mean for low expression = 7.7, SD = 8.1; mean for high expression = 5.8, SD = 4.1, *t*(114) = 1.5, *p* < .126, two-tailed], and Attentional Control Scale [mean for low expression = 51.6, SD = 9.3; mean for high expression = 51.5, SD = 9.0, *t*(114) < .1]. The genotyping groups did not differ on any of the pretraining questionnaire measures for either the positive ABM (all *t*s < 1.2) or the negative ABM (all *t*s < 1.3) group.

**Figure 1:**
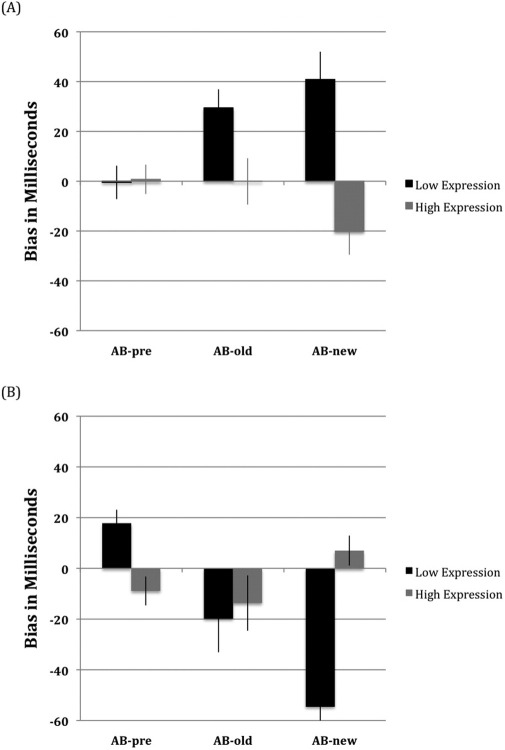
The mean bias scores for each genotyping group before attention bias modification (ABM) training and for both new and old images following ABM training for the positive **(A)** and negative **(B)** ABM training groups. AB-new, new images; AB-old, old images; AB-pre, before ABM training.

The attentional bias scores before and after ABM training is shown in Figure 1. The three-way interaction between genotype group, ABM group, and bias index was significant, *F*(2,224) = 14.8, *p* < .001, Cohen's *f* = .36. For the low-expression group, there was a significant ABM group × bias index interaction, *F*(2,120) = 13.7, *p* < .001, Cohen's *f* = .48, such that the positive bias induced by positive ABM training was larger than at pretraining for both old [*t*(30) = −3.1, *p* < .004, two-tailed, Cohen's *d* = .56] and new [*t*(30) = −2.9, *p* < .006, two-tailed, Cohen's *d* = .52] items, while negative ABM induced larger negative biases for both old [*t*(30) = 2.5, *p* < .018, two-tailed, Cohen's *d* = .45] and new [*t*(30) = 3.4, *p* < .002, two-tailed, Cohen's *d* = .60] items. For the high-expression group, while the ABM group × bias index interaction did reach significance, *F*(2,104) = 3.9, *p* < .05, Cohen's *f* = .27, follow-up comparisons revealed only trends for larger biases with new items following positive [*t*(27) = 1.9, *p* < .07, two-tailed, Cohen's *d* = .36] and negative [*t*(27) = −2.0, *p* < .06, two-tailed, Cohen's *d* = .40] ABM training.

The interaction was also examined for each ABM group separately and this showed that the attentional bias of the low-expression group was altered significantly by positive ABM for both new [*t*(31) = −2.9, *p* < .006, two-tailed, Cohen's *d* = .82] and old [*t*(31) = −3.1, *p* < .004, two-tailed, Cohen's *d* = .76] items. For this group, negative ABM also changed bias significantly in the expected direction for both new [*t*(30) = 3.4, *p* < .002, two-tailed, Cohen's *d* = .53] and old [*t*(30) = 2.5, *p* < .018, two-tailed, Cohen's *d* = .53] items. In marked contrast, the bias of the high-expression group was not modified by either positive or negative ABM interventions.

**Figure 2:**
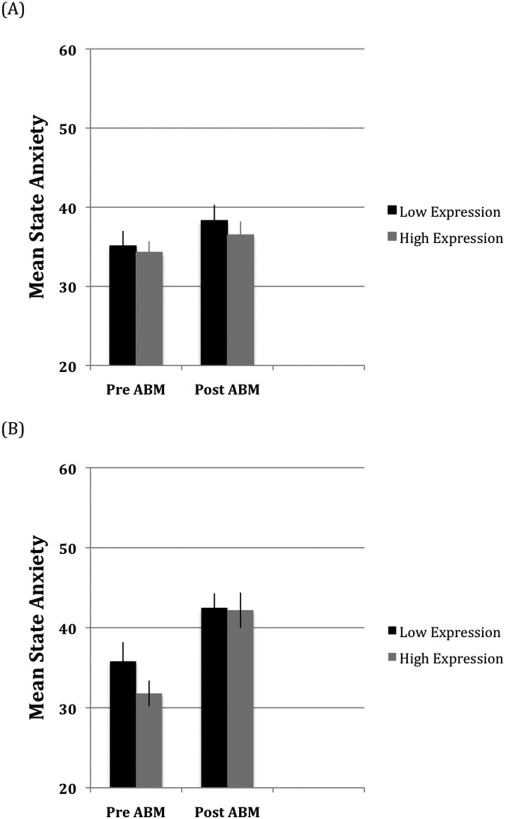
Mean self-reported state-anxiety before and after attention bias modification (ABM) for the positive **(A)** and negative **(B)** ABM training groups.

**Figure 3:**
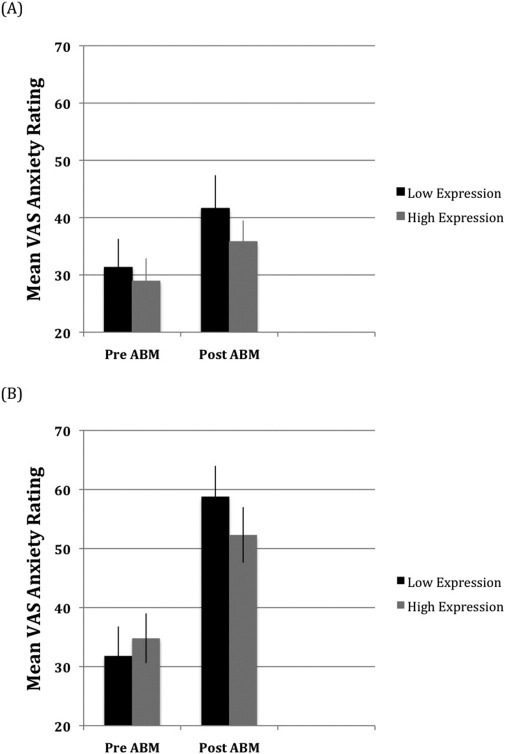
Mean scores on the visual analogue scale ratings of anxiety before and after attention bias modification (ABM) for the positive **(A)** and negative **(B)** ABM training groups. VAS, visual analogue scale.

**Figure 4:**
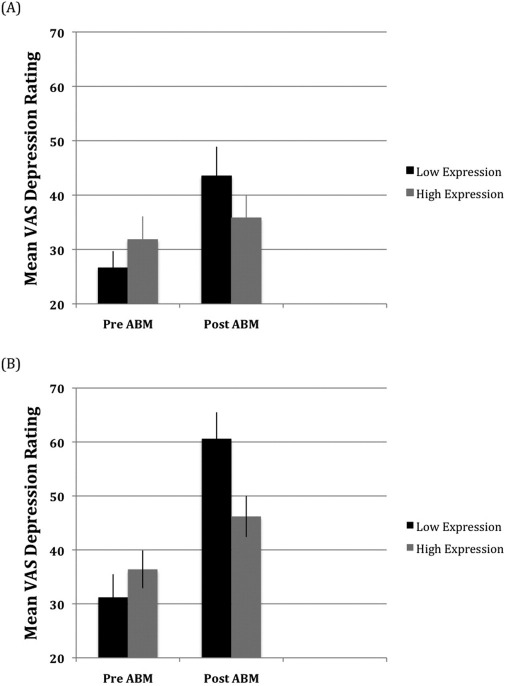
Mean scores on the visual analogue scale ratings of depression before and after attention bias modification (ABM) for the positive **(A)** and negative **(B)** ABM training groups. VAS, visual analogue scale.

Figure 2 shows the mean state-anxiety reported for each ABM group. The three-way interaction was not significant, but there was an ABM group × session interaction, *F*(1,112) = 9.3, *p* < .003, Cohen's *f* = .29, due to a larger increase in state-anxiety following training for negative ABM [*t*(58) = −2.1, *p* < .04, Cohen's *f* = .29] compared with positive ABM [*t*(56) = −5.9, *p* < .000, Cohen's *f* = .78]. The mean ratings on the VAS scales also revealed ABM group × session interactions for anxiety [Figure 3: *F*(1,112) = 8.0, *p* < .005, Cohen's *f* = .27] and depression [Figure 4: *F*(1,112) = 5.5, *p* < .021, Cohen's *f* = .22], which were not qualified by genotype group. For the anxiety ratings, the increase following training was greater for negative ABM [*t*(56) = −7.1, *p* < .001, Cohen's *d* = .94] than for positive ABM [*t*(58) = −2.4, *p* < .018, Cohen's *d* = .32]. Depression ratings also increased more following negative ABM [*t*(56) = −7.1, *p* < .001, Cohen's *d* = .95] than positive ABM [*t*(58) = −3.6, *p* < .001, Cohen's *d* = .47]. Finally, a genotype group × session interaction for the depression ratings, *F*(1,112) = 17.1, *p* < .000, Cohen's *f* = .39, was due to larger increases in depression following ABM for the low-expression [*t*(61) = −7.4, *p* < .000, Cohen's *d* = .94] relative to the high-expression [*t*(61) = −2.9, *p* < .005, Cohen's *d* = .39] genotyping group.

## Discussion

Changes in attentional bias following ABM were greater in people with low-expression relative to high-expression forms of the serotonin transporter gene. This cognitive malleability to environmental contingencies explains why s-allele carriers (i.e., low expression) are faster to learn fear and develop neural circuits that are more sensitive to threat (27,29). However, the attentional systems of s-allele carriers were also more responsive to positive ABM training, relative to long-allele carriers, providing direct support for the view that the low-expression form of the serotonin transporter gene is best conceived of as a plasticity gene rather than a vulnerability gene. The low-expressing form tunes people into the affective significance of their surroundings—whether negative or positive—moving us beyond the notion that the s allele is a risky genotype, whereas the l allele is protective. Instead, it seems that there's a cost to protective genotypes, such as a reduced ability to maximize the potential of favorable situations (2,13,15).

The impact of ABM on mood and anxiety was relatively modest. Depression and anxiety both increased following ABM, with the negative ABM generally leading to larger increases than the positive ABM. This pattern is understandable given that every trial on our ABM procedure contained a negative image. Even though participants' attention was directed away from the negative images during the positive ABM, these threat-related images are powerful cues to attention (17) and therefore are likely to be noticed over several hundred trials. For clinical interventions, ABM procedures with positive and neutral images are unlikely to have such negative effects on mood. Genotype group strongly influenced the degree of change on attentional bias, while the effects on mood were less clear-cut. This is likely due to less sensitivity of the self-report scales when compared with measures of cognitive bias (18), in combination with just a single session of ABM training. Future research should include a wider range of more sensitive outcome measures.

One implication of our results is that s-allele carriers should gain most from therapeutic interventions such as ABM. However, a recent study reported that posttraumatic stress disorder patients with the short allele showed a poorer response to cognitive-behavior therapy than did those with two long alleles (40). Thus, at 6-month follow-up, fewer patients in the l-allele group met diagnostic criteria for posttraumatic stress disorder (*n* = 2, 15%) than those in the s-allele group (*n* = 14, 48%). One possibility is that even though the cognitive systems of the short-allele carriers are more malleable, this malleability, in turn, results in a much more deeply ingrained set of biases over a lifetime. In other words, the heightened sensitivity to environmental input that occurs in s-allele carriers makes it difficult for them to overcome pre-existing biases and deeply engrained neural circuits that are particularly responsive to danger cues. By the same token, this group should, however, be ultimately more responsive to interventions such as CBT and ABM, and this is an important focus of future research. The use of procedures, like ABM, that specifically target low-level biases may prove particularly effective for this genotype.

Several limitations to our study need to be addressed in future research. Our sample size was relatively small and we examined just one genetic polymorphism. These findings should be replicated with a larger sample and a greater range of potential genetic predictors (e.g., the catechol-*O*-methyltransferase Valine158Methionine polymorphism that is also linked to fear learning). A direct and sensitive measure of stress reactivity after the intervention should also be included in future studies. It would also be useful for future research to include more conventional ABM paradigms that include neutral, in addition to positive and negative, material. Even though this does confound arousal and valence, the absence of negative images in positive training is likely to prevent the negative effect on mood we saw here.

Our results present the first evidence that variation on the serotonin transporter polymorphism leads to different degrees of sensitivity to short-term interventions that modify fundamental biases in cognitive processing. This provides a potential mechanism through which those with the low-expression form of this gene are more susceptible to environmental events for better and for worse. Our results imply that those with the low-expression form may benefit most from interventions aimed at reducing toxic biases in attention, even though they may have more deeply ingrained biases.
